# Achieving sustainable health equity

**DOI:** 10.2471/BLT.21.286523

**Published:** 2021-11-25

**Authors:** Arachu Castro, Michael Marmot, Juan Garay, Armando de Negri, Paulo Buss, on behalf of the Sustainable Health Equity Movement

**Affiliations:** a Department of International Health and Sustainable Development, Tulane University School of Public Health and Tropical Medicine, 1440 Canal Street, New Orleans, LA 70112 United States of America.; b Department of Epidemiology and Public Health, University College London Institute of Health Equity, London, England.; c National School of Public Health of Spain, Madrid, Spain.; d World Social Forum on Health and Social Security, Porto Alegre, Brazil.; e Oswaldo Cruz Foundation, Rio de Janeiro, Brazil.

To achieve sustainable health equity, we need to address two intertwined challenges: social inequality and the climate crisis. They both create health inequalities within and between countries; when these health inequalities are avoidable and unjust, they are known as health inequities. Building on the extensive knowledge and resources that already exist to sustainably improve health for everyone, the Sustainable Health Equity Movement^1^ aims to promote sustainable health equity as an ethical principle that guides all national and international economic, social and environmental policies.^2^

The unequal distribution of power, money and other resources within and between countries, environmental damage, and political and armed conflict are structural drivers of inequities and the source of health inequities across the social gradient.^3^^,^^4^ Creating the conditions for all people and future generations to attain their best health potential and lead dignified lives requires taking global, national and local actions on the structural and daily life factors (that is, the social determinants of health),^5^ with good governance based on human rights approaches.

## Inequities are unsustainable

Inequities are present across a spectrum of issues. The existence of large health inequities indicates that the basic needs of many population groups are not being met, whereas the world has sufficient economic resources to ensure that these needs are met for all. Exposure to environmental risks is inequitably increasing due to air and water pollution, food contamination, epidemics, pandemics and global warming, while access to health services is skewed against those in greatest need. The right to opportunity and fulfilment of one’s capabilities is often unmet by lack of continuing education throughout life and spaces for self-fulfilment, creativity and innovation. As a result of all these inequities, and despite progress in narrowing inequality gaps in child mortality and some other health indicators, excess premature mortality remains high.^6^ The World Health Organization Commission on Social Determinants of Health^5^ called for attention to the root causes of health inequity and to measure its levels, distribution and trends.

Education is a vital resource for health across generations. However, sharing knowledge globally is also important. Humankind generates a massive amount of new knowledge every day. People thrive on challenges, continuous education and development, imagination, collaboration, technological means and resources. When applied to national or global challenges with a sustainable health equity approach, knowledge contributes to a shared aspiration and, if accessible for all, becomes a national and global public good. While global sharing is growing, international markets often drive knowledge, especially in relation to health products, through patent monopolies. These monopolies generate profits for some and preclude access to those who need it the most, increasing the burden of inequity. The coronavirus disease 2019 (COVID-19) pandemic reveals the imbalance of the global market framework and its inability to alone provide solutions to health issues – also shown during the first two decades of the human immunodeficiency virus global epidemic. To achieve equity in knowledge, developing an internationally binding framework that promotes and protects the main principles of global public goods, such as universal immunization, is crucial. Such a framework would set more ambitious targets on health equity under the sustainable development goals (SDGs). 

Through people’s labour, knowledge transforms natural resources into technology, products and services. However, current economic arrangements have several problems. With the decline of trade union movements, workers have lost the power to improve working conditions and receive fair remuneration. The returns on capital have progressively increased, while rewards to most workers have shrunk, and new technologies have meant that many people across different economic sectors are excluded from the job market.

These economic arrangements result in the growth of inequalities in income and wealth in many countries. First, economic power is concentrated among fewer individuals who have disproportionate economic and political power. Second, with the economy’s increasing financialization, there has been a shift away from productive investment towards currency speculation, trade in financial derivatives and taking value out of corporations rather than re-investment. While global poverty had been falling until 2020, the COVID-19 pandemic has reversed this trend – yet the very rich have increased their wealth. Third, economic growth has become the marker of social success, regardless of ecological and climate damage, and distribution of income and wealth. To achieve greater economic equity, governments need to adopt a political economy approach aimed at redistributing accumulated wealth through fair tax systems and universal and equitable public social protection systems. Health equity should be the main goal. When controlled by democratic mechanisms, a restructuring of the economy can prevent the financialization of social policies, financial speculation and excess economic accumulation; guarantee a sufficient and fair basic income; and establish global, regional and national frameworks of fiscal and territorial equity, and more ambitious targets on health equity under SDG 10 (reduce inequality within and among countries).

We survive and thrive by using natural resources for our vital needs, well-being, technology and the advancement of knowledge. The way we use natural resources, driven by constant economic growth, global scale production, trade and urban consumption and lifestyles has upset the balance to the detriment of nature. This unbalance has been well documented in the literature of planetary boundaries, which concludes that carbon emissions, fresh water depletion and loss of biodiversity are already beyond nature’s regeneration capacity.^7^ The overuse of natural resources in the past two centuries has caused global warming and is having a direct effect on the health of people and on the environment, with a detrimental result for the current generation and for generations to come, determining intergenerational inequity.

Global warming has been and continues to be caused by those who are better protected from its consequences, reflecting all inequities, with higher risk and higher burden among those who contributed the least to climate change. Forecasts predict that the life expectancy of the generation born in the 21st century will be, for the first time since data are recorded, lower than that of the previous generation.^8^ Since the Kyoto Protocol entered into effect in 2005, the world has managed to reduce the rate of growth of carbon emissions, but we are far from the levels that would prevent the point of no return of 1.5 °C above pre-industrial levels. To achieve environmental equity, guaranteeing the respect of planetary boundaries at the individual, national and global levels, and charting the transition to a post-fossil fuels era with an enhanced attention and more ambitious targets on health equity under SDG 13 are needed.

## Taking action

Public institutions with good governance decide economic, social and environmental policies, manage public resources and deliver essential services in a manner that promotes human rights and accountability^9^ and that respond to millions of people who claim a fairer, more equitable world. The right to health, a fundamental human right indispensable for the exercise of other human rights, is recognized in numerous international instruments,^10^ some of which include components that are legally enforceable. The International Covenant on Economic, Social and Cultural Rights provides the most comprehensive article on the right to health in international human rights law and is the only international legally binding framework related to health equity. The covenant, which recognizes the right of all to enjoy the highest attainable standard of physical and mental health, is ratified by 171 countries and signed by another four.^11^ However, the attention to equity is indirect, limited and not monitored, and it relies on a concept of progressiveness according to the available resources in each country that disregards the imbalance of international relationships. The implementation of the covenant requires significant commitments from governments to provide universal access to health services, stop actively violating human rights, decrease spending in areas that do not prioritize sustainable health equity and increase resources in those areas that meet the obligations of countries under the covenant. 

To achieve sustainable health equity, the Sustainable Health Equity Movement calls for several actions. First, promoting sustainable health equity as an ethical and human rights principle that guides all national and international economic, social and environmental policies, including through national and international legal frameworks to enhance accountability and implementation of the right to health. Second, creating an independent Sustainable Health Equity Commission, with a Rapporteur who would monitor progress and report to the United Nations (UN) Secretary-General to issue recommendations. The establishment of such a commission could be achieved through a UN General Assembly resolution codified in a protocol to the covenant, along with the right to a life with dignity for all, based on individual and collective equity, as well as inclusion in a future convention on the right to development.^12^ The Rapporteur would work alongside the Human Rights Commissioner and the Special Rapporteur on the Right to Health and align with other legal frameworks that contribute to sustainable health equity such as economic, social, cultural, disability and children’s rights, gender equity and migrant health. Bringing all these arguments together adds value, as it does so in a concerted way. Finally, promoting collaborations with scientists and activists to gather evidence on the burden of unsustainable health inequity and demand greater accountability from governments would advance sustainable health equity.

## References

[1] Sustainable health equity movement [Internet]. 2021. Available from: https://www.sustainablehealthequity.org/ [cited 2021 Jun 1].

[2] Chiriboga D, Garay J, Buss P, Madrigal RS, Rispel LC. Health inequity during the COVID-19 pandemic: a cry for ethical global leadership. Lancet. 2020 05 30;395(10238):1690–1. 10.1016/S0140-6736(20)31145-4 32419711 PMC7225689

[3] Just societies - health equity and dignified liv–s - Report of the Commission of the Pan American Health Organization on Equity and Health Inequalities in the Americas. Washington, DC: Pan American Health Organization; 2019. Available from: https://iris.paho.org/handle/10665.2/51571 [cited 2021 Jun 1].

[4] Build back fairer: achieving health equity in the Eastern Mediterranean Region: report of the Commission on Social Determinants of Health in the Eastern Mediterranean Region – executive summary. Cairo: World Health Organization Regional Office for the Eastern Mediterranean; 2021. Available from: https://applications.emro.who.int/docs/9789290224976-eng.pdf?ua=1 [cited 2021 Jun 1].

[5] Closing the gap in a generation: health equity through action on the social determinants of health. Geneva: World Health Organization; 2005. Available from: https://www.who.int/social_determinants/final_report/csdh_finalreport_2008.pdf [cited 2021 Jun 1].

[6] Clark H, Coll-Seck AM, Banerjee A, Peterson S, Dalglish SL, Ameratunga S, et al. A future for the world’s children? A WHO-UNICEF-Lancet Commission. Lancet. 2020 Feb 22;395(10224):605–58. 10.1016/S0140-6736(19)32540-1 10.1016/S0140-6736(19)32540-1 32085821

[7] Steffen W, Richardson K, Rockström J, Cornell SE, Fetzer I, Bennett EM, et al. Planetary boundaries: guiding human development on a changing planet. Science. 2015 Feb 13;347(6223):1259855. 10.1126/science.1259855 25592418

[8] Harper S, Riddell CA, King NB. Declining life expectancy in the United States: missing the trees for the forest. Annu Rev Public Health. 2021 Apr 1;42(1):381–403. 10.1146/annurev-publhealth-082619-104231 33326297

[9] Governance for sustainable development: integrating governance in the post-2015 development framework. New York: United Nations Development Programme; 2014. Available from: https://www.undp.org/publications/discussion-paper-governance-sustainable-development [cited 2021 Jun 1].

[10] Universal declaration of human rights. Article 25.1. New York: United Nations; 1948. Available from: https://www.un.org/en/udhrbook/pdf/udhr_booklet_en_web.pdf [cited 2021 Jun 1].

[11] International covenant on economic, social and cultural rights (ICESCR). New York: United Nations; 1966. Available from: https://treaties.un.org/Pages/ViewDetails.aspx?src=IND&mtdsg_no=IV-3&chapter=4 [cited 2021 Jun 1].

[12] Resolution A/RES/41/128. Declaration on the right to development. United Nations General Assembly, New York, 4 December 1986. New York: United Nations; 1986. Available from: https://www.ohchr.org/Documents/Issues/Development/DeclarationRightDevelopment_en.pdf [cited 2021 Jun 1].

